# 4-(2-Benzoyl­benzoyl)-*N*,*N*-diphenyl­aniline

**DOI:** 10.1107/S160053681202466X

**Published:** 2012-06-13

**Authors:** P. Narayanan, K. Sethusankar, M. Nandakumar, A. K. Mohanakrishnan

**Affiliations:** a Department of Physics, RKM Vivekananda College (Autonomous), Chennai 600 004, India; b Department of Organic Chemistry, University of Madras, Maraimalai Campus, Chennai 600 025, India

## Abstract

The asymmetric unit of the title compound, C_32_H_23_NO_2_, comprises two crystallographically independent mol­ecules. In both mol­ecules, the geometries about the N atoms deviate significantly from the ideal trigonal–planar geometry with bond-angle sums about the N atom of 359.32° in one mol­ecule and 359.86° in the other. The O atoms of the carbonyl groups are deviated significantly from the central benzene rings by 0.6747 (14) and −1.1223 (13) Å in one molecule and −0.6230 (13) and 1.1559 (12) Å in the other. In the diphenyl­aniline units, the terminal phenyl rings are almost orthogonal to each other, with dihedral angles of 89.79 (9) and 89.76 (9)°. The crystal structure features C—H⋯O and C—H⋯π inter­actions.

## Related literature

For the biological importance and usage of diketones, see: Sugawara *et al.* (2001 ▶); Kennedy *et al.* (2002 ▶); Song *et al.* (2006 ▶); Kakimoto *et al.* (2008 ▶). For related structures, see: Narayanan *et al.* (2011 ▶); Wu *et al.* (2011 ▶).
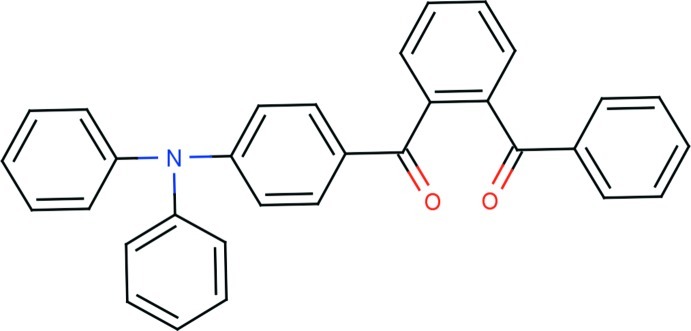


## Experimental

### 

#### Crystal data

C_32_H_23_NO_2_
*M* *_r_* = 453.51
Triclinic, 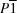

*a* = 10.7599 (3) Å
*b* = 13.0389 (3) Å
*c* = 17.9453 (5) Å
α = 90.447 (2)°
β = 98.415 (2)°
γ = 108.904 (2)°
*V* = 2352.13 (11) Å^3^
*Z* = 4
Mo *K*α radiation
μ = 0.08 mm^−1^
*T* = 295 K
0.30 × 0.25 × 0.20 mm

#### Data collection

Bruker Kappa APEXII CCD diffractometer
Absorption correction: multi-scan (*SADABS*; Bruker, 2008 ▶) *T*_min_ = 0.977, *T*_max_ = 0.984
39694 measured reflections
8280 independent reflections
6006 reflections with *I* > 2σ(*I*)
*R*_int_ = 0.030

#### Refinement

*R*[*F* ^2^ > 2σ(*F* ^2^)] = 0.036
*wR*(*F* ^2^) = 0.097
*S* = 1.01
8280 reflections
631 parameters
H-atom parameters constrained
Δρ_max_ = 0.18 e Å^−3^
Δρ_min_ = −0.17 e Å^−3^

### 

Data collection: *APEX2* (Bruker, 2008 ▶); cell refinement: *SAINT* (Bruker, 2008 ▶); data reduction: *SAINT* (Bruker, 2008 ▶); program(s) used to solve structure: *SHELXS97* (Sheldrick, 2008 ▶); program(s) used to refine structure: *SHELXL97* (Sheldrick, 2008 ▶); molecular graphics: *ORTEP-3* (Farrugia, 1997 ▶); software used to prepare material for publication: *SHELXL97* and *PLATON* (Spek, 2009 ▶).

## Supplementary Material

Crystal structure: contains datablock(s) global, I. DOI: 10.1107/S160053681202466X/rk2362sup1.cif

Structure factors: contains datablock(s) I. DOI: 10.1107/S160053681202466X/rk2362Isup2.hkl

Supplementary material file. DOI: 10.1107/S160053681202466X/rk2362Isup3.cml

Additional supplementary materials:  crystallographic information; 3D view; checkCIF report

## Figures and Tables

**Table 1:** Hydrogen-bond geometry (Å, °) *Cg*1 and *Cg*2 are the centroids of the C8–C13 and C27′–C32′ phenyl rings, respectively.

*D*—H⋯*A*	*D*—H	H⋯*A*	*D*⋯*A*	*D*—H⋯*A*
C31′—H31′⋯O2^i^	0.93	2.51	3.419 (2)	166
C16—H16⋯O2′	0.93	2.44	3.219 (2)	141
C30′—H30′⋯*Cg*1^i^	0.93	3.00	3.894 (2)	162
C4′—H4′⋯*Cg*2^ii^	0.93	2.95	3.731 (2)	142

Symmetry codes: (i) 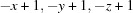; (ii) 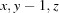.

**Fig. 1.**
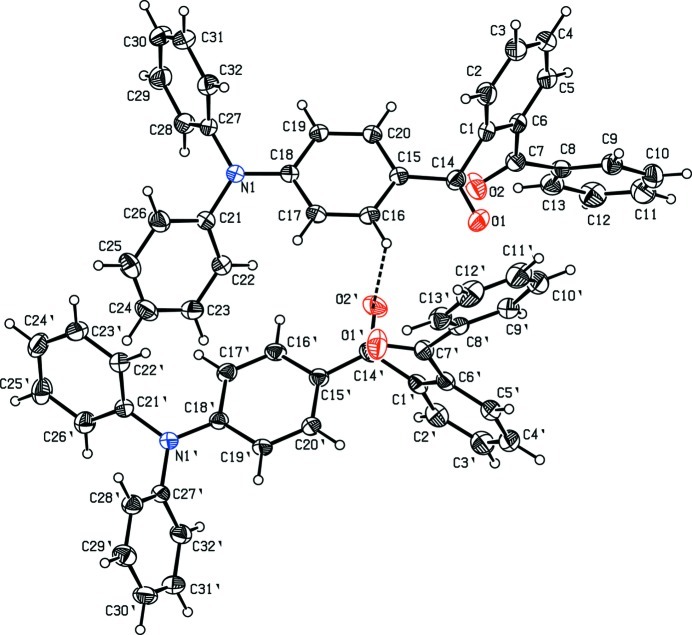
The asymmetric unit of the title compound with the atom numbering scheme. Displacement ellipsoids are drawn at 30% probability level. The H atoms are presented as a small spheres of arbitrary radius.

**Fig. 2.**
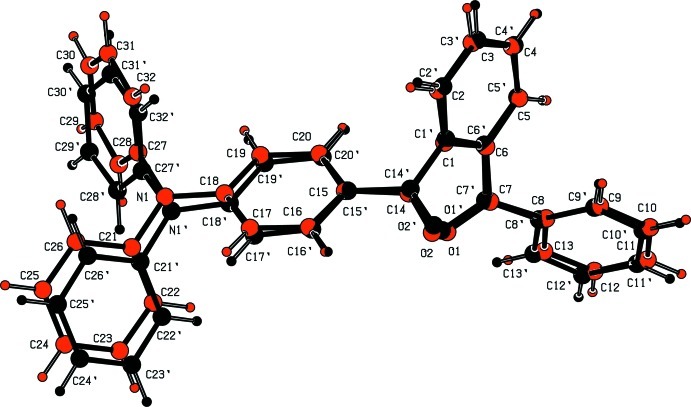
The molecule 1 (Red) and molecule 2 (Black) of the title compound overlapping with each other. H atoms are shown as spheres of arbitary radius.

**Fig. 3.**
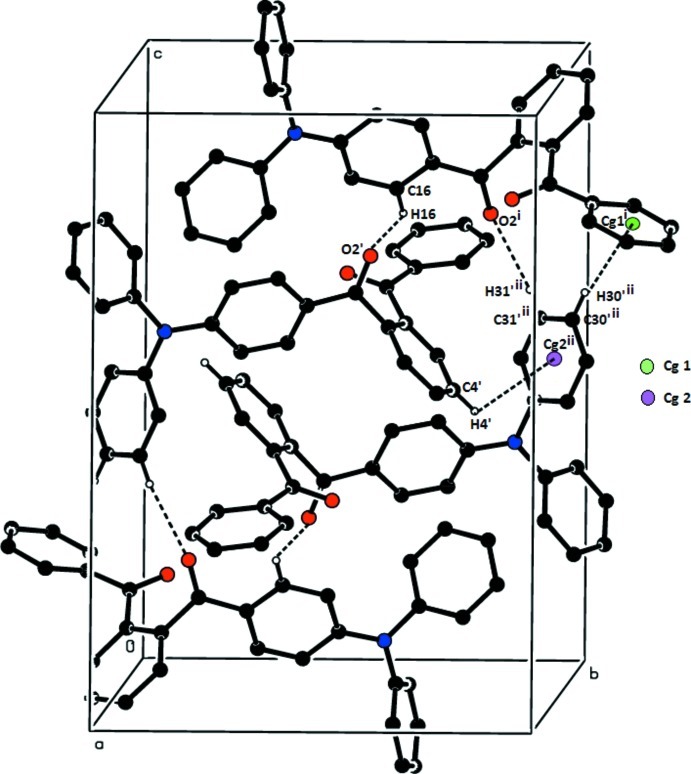
The crystal packing of the title compound, viewed down c axis, showing intermolecular C–H···O and C–H···π hydrogen bonds (dashed lines). Symmetry codes: (i) -x+1, -y+1, -z+1; (ii) x, y-1, z.

## supplementary crystallographic information

### Comment

The cyclic ketones play a significant role in increasing the red blood cells. They are also useful as outstanding hematopoietic agents in medicine, in particular, in the treatment of cancer chemotherapy, radiotherapy and drugtherapy (Sugawara *et al.*, 2001). Triarylamine based organic semiconductors have been intensively investigated as hole transport materials for electrooptic devices like Organic Field Effect Transistor with good mobility and high on/off ratio (Kennedy *et al.*, 2002, Song *et al.*, 2006) and Organic Light Emitting Diode materials (Kakimoto *et al.*, 2008). In view of this background the current study is undertaken and the structure of the compound is solved.

X-Ray analysis confirms the molecular structure and atom connectivity as illustrated in Fig. 1. The title compound, C_32_H_23_NO_2_, comprises two crystallographically independent molecules in the asymmetric unit. The corresponding bond lengths and bond angles of both the molecules agree well with each other as illustrated in the overlapping diagram (Fig. 2).

The bond angles around nitrogen atoms N1 and N1' (C18-N1-C21 = 121.97 (12)°, C18-N1-C27 = 119.64 (12)°, C21-N1-C27 = 117.71 (12)° & C18'-N1'-C21' = 120.84 (12)°, C18'-N1'-C27' = 121.29 (12)°, C21'-N1'-C27' = 117.73 (12)°). are significantly deviated from the ideal trigonal geometry value (120°). The terminal phenyl rings of the diphenylaniline moieties, (C21-C26), (C27-C32) and (C21'-C26'), (C27'-C32') are almost perpendicular to each other, with the dihedral angles between them being 89.79 (9)° and 89.76 (9)°, respectively.

The oxygen atoms of carbonyl groups are significantly deviated (O1 = 0.6747 (14)Å, O2 = -1.1223 (13)Å & O1' = -0.6230 (13)Å, O2' = 1.1559 (12)Å) from the central phenyl rings (C1-C6) & (C1'-C6'), respectively. The central phenyl rings in both the molecules, (C1-C6) and (C1'-C6') form the dihedral angles of 56.25 (9)° and 55.67 (8)°, with the leftside phenyl rings (C8-C13) and (C8'-C13'), respectively. The central phenyl rings (C1-C6) and (C1'-C6') in both the molecules form the dihedral angles of 80.52 (8)° and 88.40 (8)° with the rightside phenyl rings (C15-C20) and (C15'-C20'), respectively, which shows that the central phenyl rings are almost orthogonal with the phenyl rings in both the molecules. The title compound exibits the structural similarities with other already reported related structures (Narayanan *et al.*, 2011; Wu *et al.*, 2011).

The crystal packing is stabilized by intermolecular C16–H16···O2', C31'–H31'···O2^i^, C30–H30···*Cg*1^i^ and C4'–H4'···*Cg*2^ii^ interactions. *Cg*1 is the centre of gravity of the phenyl ring (C8-C13) and *Cg*2 is the centre of gravity of the phenyl ring (C27'-C32') (Table 1). The symmetry codes: (i) 1-*x*, 1-*y*, 1-*z*; (ii) *x*, -1+*y*, *z*. The packing view of the title compound is shown in Fig. 3.

### Experimental

To a solution of benzo[*c*]furan (0.52 g, 1.19 mmol) in *DCM* (15 ml), *m*-*CPBA* (0.40 g, 1.78 mmol) was added and the reaction mixture was stirred at room temperature for 5 minutes. It was then poured into saturated sodium bicarbonate solution, extracted with *DCM* (3×30 ml). The combined organic extract was washed with water (2×30 ml) and dried (Na_2_SO_4_). Removal of solvent followed by column chromatographic purification (silica gel, 5% *EA*/Hexane) afforded the diketone as a pale yellow solid with the yield of (0.44 g, 81%). The product was dissolved in chloroform and heated for two minutes. The resulting solution was subjected to crystallization by slow evaporation of the solvent resulting in single crystals suitable for XRD studies. M.p. 447-448 K.

^1^H NMR (300 MHz, CDCl_3_): δ 7.66 (d,J = 7.2 Hz, 2H, *Ar*H), 7.55-7.45 (m, 7H, *Ar*H), 7.35-7.30 (m, 2H, *Ar*H), 7.26-7.19 (m, 5H, *Ar*H), 7.07- 7.04 (m, 5H, *Ar*H), 6.82 (d,J = 8.7 Hz, 2H, *Ar*H). ^13^C NMR (75 MHz, CDCl_3_): δ 196.8, 194.9, 152.2, 146.4, 140.7, 139.8, 137.3, 133.0, 131.6, 130.3, 129.9, 129.87, 129.63, 129.53, 129.38, 128.3, 126.1, 124.8, 119.4. DEPT. 135 (75 MHz, CDCl_3_): δ 133.0, 131.6, 130.3, 129.94, 129.87, 129.63, 129.53, 129.38, 128.3, 126.1, 124.8, 119.4.

### Refinement

The positions of hydrogen atoms were localized from the difference electron density maps and their distances were geometrically constrained. The H atoms bound to the C atoms were treated as riding atoms, with d(C–H) = 0.93Å and *U*_iso_(H) = 1.2*U*_eq_(C).

### Crystal data

**Table:**

C_32_H_23_NO_2_	*Z* = 4
*M**_r_* = 453.51	*F*(000) = 952
Triclinic, *P*1	*D*_x_ = 1.281 Mg m^−^^3^
Hall symbol: -P 1	Melting point = 447–448 K
*a* = 10.7599 (3) Å	Mo *K*α radiation, λ = 0.71073 Å
*b* = 13.0389 (3) Å	Cell parameters from 8280 reflections
*c* = 17.9453 (5) Å	θ = 1.2–25.0°
α = 90.447 (2)°	µ = 0.08 mm^−^^1^
β = 98.415 (2)°	*T* = 295 K
γ = 108.904 (2)°	Block, yellow
*V* = 2352.13 (11) Å^3^	0.30 × 0.25 × 0.20 mm

### Data collection

**Table:**

Bruker Kappa APEXII CCD diffractometer	8280 independent reflections
Radiation source: fine-focus sealed tube	6006 reflections with *I* > 2σ(*I*)
Graphite monochromator	*R*_int_ = 0.030
φ & ω scans	θ_max_ = 25.0°, θ_min_ = 1.2°
Absorption correction: multi-scan (*SADABS*; Bruker, 2008)	*h* = −12→12
*T*_min_ = 0.977, *T*_max_ = 0.984	*k* = −15→15
39694 measured reflections	*l* = −21→21

### Refinement

**Table:**

Refinement on *F*^2^	Primary atom site location: structure-invariant direct methods
Least-squares matrix: full	Secondary atom site location: difference Fourier map
*R*[*F*^2^ > 2σ(*F*^2^)] = 0.036	Hydrogen site location: inferred from neighbouring sites
*wR*(*F*^2^) = 0.097	H-atom parameters constrained
*S* = 1.01	*w* = 1/[σ^2^(*F*_o_^2^) + (0.0402*P*)^2^ + 0.4791*P*] where *P* = (*F*_o_^2^ + 2*F*_c_^2^)/3
8280 reflections	(Δ/σ)_max_ < 0.001
631 parameters	Δρ_max_ = 0.18 e Å^−^^3^
0 restraints	Δρ_min_ = −0.17 e Å^−^^3^

### Special details

**Table:**

Geometry. All s.u.'s (except the s.u. in the dihedral angle between two l.s. planes) are estimated using the full covariance matrix. The cell s.u.'s are taken into account individually in the estimation of s.u.'s in distances, angles and torsion angles; correlations between s.u.'s in cell parameters are only used when they are defined by crystal symmetry. An approximate (isotropic) treatment of cell s.u.'s is used for estimating s.u.'s involving l.s. planes.
Refinement. Refinement of *F*^2^ against ALL reflections. The weighted *R*-factor *wR* and goodness of fit *S* are based on *F*^2^, conventional *R*-factors *R* are based on *F*, with *F* set to zero for negative *F*^2^. The threshold expression of *F*^2^ > σ(*F*^2^) is used only for calculating *R*-factors(gt) *etc*. and is not relevant to the choice of reflections for refinement. *R*-factors based on *F*^2^ are statistically about twice as large as those based on *F*, and *R*-factors based on ALL data will be even larger.

### Fractional atomic coordinates and isotropic or equivalent isotropic displacement parameters (Å^2^)

**Table:**

	*x*	*y*	*z*	*U*_iso_*/*U*_eq_	
C1	0.27625 (16)	0.07397 (12)	0.07392 (9)	0.0430 (4)	
C1'	0.69995 (15)	0.41476 (12)	0.42657 (9)	0.0438 (4)	
C2	0.36460 (18)	0.08266 (14)	0.02410 (10)	0.0551 (4)	
H2	0.4403	0.1438	0.0279	0.066*	
C2'	0.60717 (17)	0.36678 (14)	0.47244 (10)	0.0570 (4)	
H2'	0.5282	0.3826	0.4678	0.068*	
C3	0.34265 (19)	0.00215 (14)	−0.03134 (10)	0.0603 (5)	
H3	0.4038	0.0089	−0.0642	0.072*	
C3'	0.62992 (19)	0.29579 (15)	0.52498 (11)	0.0629 (5)	
H3'	0.5659	0.2634	0.5549	0.076*	
C4'	0.74661 (19)	0.27278 (14)	0.53322 (10)	0.0581 (5)	
H4'	0.7624	0.2254	0.5690	0.070*	
C4	0.23104 (19)	−0.08763 (14)	−0.03807 (10)	0.0579 (5)	
H4	0.2158	−0.1417	−0.0757	0.069*	
C5'	0.84025 (17)	0.32012 (12)	0.48821 (9)	0.0489 (4)	
H5'	0.9196	0.3048	0.4942	0.059*	
C5	0.14133 (17)	−0.09762 (13)	0.01107 (9)	0.0510 (4)	
H5	0.0655	−0.1587	0.0062	0.061*	
C6	0.16218 (15)	−0.01811 (12)	0.06770 (8)	0.0425 (4)	
C6'	0.81831 (15)	0.39033 (11)	0.43398 (8)	0.0412 (4)	
C7'	0.92194 (16)	0.44636 (13)	0.38769 (9)	0.0459 (4)	
C7	0.06076 (16)	−0.02446 (13)	0.11750 (9)	0.0465 (4)	
C8'	1.01657 (15)	0.39358 (12)	0.36670 (8)	0.0429 (4)	
C8	−0.02619 (15)	−0.13156 (12)	0.13754 (8)	0.0436 (4)	
C9	0.01631 (17)	−0.22103 (13)	0.14645 (9)	0.0495 (4)	
H9	0.0995	−0.2173	0.1356	0.059*	
C9'	0.98162 (17)	0.28211 (13)	0.35386 (9)	0.0500 (4)	
H9'	0.8988	0.2370	0.3625	0.060*	
C10'	1.0691 (2)	0.23776 (16)	0.32837 (10)	0.0632 (5)	
H10'	1.0440	0.1631	0.3186	0.076*	
C10	−0.0645 (2)	−0.31547 (14)	0.17138 (10)	0.0625 (5)	
H10	−0.0346	−0.3746	0.1784	0.075*	
C11'	1.1919 (2)	0.30246 (19)	0.31746 (11)	0.0741 (6)	
H11'	1.2506	0.2720	0.3006	0.089*	
C11	−0.1883 (2)	−0.32275 (16)	0.18595 (12)	0.0725 (6)	
H11	−0.2423	−0.3867	0.2028	0.087*	
C12'	1.2291 (2)	0.41293 (19)	0.33146 (12)	0.0736 (6)	
H12'	1.3136	0.4570	0.3250	0.088*	
C12	−0.2329 (2)	−0.23545 (17)	0.17560 (11)	0.0712 (5)	
H12	−0.3179	−0.2408	0.1841	0.085*	
C13'	1.14175 (18)	0.45860 (15)	0.35504 (10)	0.0586 (5)	
H13'	1.1668	0.5336	0.3632	0.070*	
C13	−0.15126 (18)	−0.14003 (15)	0.15261 (10)	0.0574 (4)	
H13	−0.1808	−0.0805	0.1472	0.069*	
C14'	0.66441 (16)	0.48548 (13)	0.36702 (9)	0.0482 (4)	
C14	0.31268 (15)	0.16201 (12)	0.13560 (9)	0.0448 (4)	
C15	0.31416 (14)	0.27125 (11)	0.11440 (8)	0.0391 (3)	
C15'	0.67316 (14)	0.59654 (12)	0.38928 (8)	0.0403 (3)	
C16	0.38144 (15)	0.36045 (12)	0.16431 (8)	0.0433 (4)	
H16	0.4286	0.3509	0.2099	0.052*	
C16'	0.61718 (15)	0.65595 (12)	0.33932 (8)	0.0444 (4)	
H16'	0.5685	0.6226	0.2933	0.053*	
C17'	0.63145 (15)	0.76188 (12)	0.35563 (8)	0.0438 (4)	
H17'	0.5914	0.7990	0.3212	0.053*	
C17	0.37950 (16)	0.46233 (12)	0.14749 (8)	0.0458 (4)	
H17	0.4279	0.5211	0.1811	0.055*	
C18'	0.70580 (14)	0.81510 (12)	0.42364 (8)	0.0382 (3)	
C18	0.30607 (15)	0.47927 (11)	0.08079 (8)	0.0410 (4)	
C19	0.24269 (16)	0.39050 (12)	0.02941 (8)	0.0455 (4)	
H19	0.1964	0.4000	−0.0166	0.055*	
C19'	0.75877 (15)	0.75522 (12)	0.47512 (8)	0.0415 (4)	
H19'	0.8059	0.7880	0.5216	0.050*	
C20'	0.74238 (15)	0.64827 (12)	0.45818 (8)	0.0422 (4)	
H20'	0.7783	0.6097	0.4936	0.051*	
C20	0.24795 (15)	0.28909 (12)	0.04605 (8)	0.0440 (4)	
H20	0.2062	0.2312	0.0107	0.053*	
C21'	0.70246 (16)	0.99230 (12)	0.37988 (8)	0.0435 (4)	
C21	0.31513 (16)	0.66186 (12)	0.12420 (8)	0.0439 (4)	
C22'	0.76141 (18)	0.99793 (13)	0.31616 (9)	0.0528 (4)	
H22'	0.8188	0.9589	0.3117	0.063*	
C22	0.26337 (18)	0.63550 (13)	0.18995 (9)	0.0534 (4)	
H22	0.2146	0.5639	0.1968	0.064*	
C23'	0.7352 (2)	1.06155 (14)	0.25908 (10)	0.0630 (5)	
H23'	0.7741	1.0645	0.2158	0.076*	
C23	0.2839 (2)	0.71546 (16)	0.24576 (10)	0.0632 (5)	
H23	0.2505	0.6970	0.2905	0.076*	
C24	0.35260 (19)	0.82138 (15)	0.23590 (10)	0.0616 (5)	
H24	0.3664	0.8747	0.2738	0.074*	
C24'	0.6521 (2)	1.12055 (14)	0.26564 (11)	0.0652 (5)	
H24'	0.6339	1.1627	0.2267	0.078*	
C25	0.40083 (18)	0.84821 (14)	0.17000 (11)	0.0637 (5)	
H25	0.4460	0.9204	0.1625	0.076*	
C25'	0.59638 (18)	1.11734 (15)	0.32946 (12)	0.0639 (5)	
H25'	0.5418	1.1588	0.3345	0.077*	
C26	0.38310 (18)	0.76933 (13)	0.11457 (10)	0.0575 (4)	
H26	0.4173	0.7885	0.0701	0.069*	
C26'	0.62057 (17)	1.05289 (13)	0.38663 (10)	0.0543 (4)	
H26'	0.5815	1.0504	0.4298	0.065*	
C27	0.24811 (17)	0.60229 (11)	−0.00952 (8)	0.0443 (4)	
C27'	0.77752 (15)	0.97481 (12)	0.51332 (8)	0.0411 (4)	
C28	0.12612 (18)	0.61642 (13)	−0.02520 (9)	0.0527 (4)	
H28	0.0764	0.6150	0.0133	0.063*	
C28'	0.89213 (16)	1.06300 (13)	0.52478 (9)	0.0476 (4)	
H28'	0.9353	1.0901	0.4843	0.057*	
C29'	0.94322 (17)	1.11138 (15)	0.59632 (10)	0.0575 (4)	
H29'	1.0202	1.1716	0.6039	0.069*	
C29	0.0772 (2)	0.63275 (14)	−0.09784 (11)	0.0644 (5)	
H29	−0.0054	0.6424	−0.1085	0.077*	
C30'	0.88069 (19)	1.07092 (16)	0.65619 (10)	0.0601 (5)	
H30'	0.9159	1.1032	0.7044	0.072*	
C30	0.1508 (2)	0.63475 (15)	−0.15438 (10)	0.0679 (5)	
H30	0.1176	0.6451	−0.2035	0.081*	
C31	0.2729 (2)	0.62168 (15)	−0.13894 (10)	0.0669 (5)	
H31	0.3226	0.6237	−0.1775	0.080*	
C31'	0.7665 (2)	0.98309 (15)	0.64515 (9)	0.0597 (5)	
H31'	0.7243	0.9556	0.6859	0.072*	
C32	0.32234 (19)	0.60559 (13)	−0.06637 (9)	0.0560 (4)	
H32	0.4055	0.5970	−0.0558	0.067*	
C32'	0.71396 (18)	0.93544 (13)	0.57353 (9)	0.0520 (4)	
H32'	0.6355	0.8766	0.5659	0.062*	
O1	0.04810 (13)	0.05895 (10)	0.14103 (8)	0.0695 (4)	
O1'	0.92974 (13)	0.53640 (10)	0.36682 (8)	0.0748 (4)	
O2	0.34835 (13)	0.14309 (9)	0.20021 (7)	0.0654 (3)	
N1'	0.72530 (14)	0.92462 (10)	0.43903 (7)	0.0465 (3)	
N1	0.29685 (15)	0.58160 (10)	0.06558 (7)	0.0504 (3)	
O2'	0.62324 (14)	0.44734 (10)	0.30249 (7)	0.0770 (4)	

### Atomic displacement parameters (Å^2^)

**Table:**

	*U*^11^	*U*^22^	*U*^33^	*U*^12^	*U*^13^	*U*^23^
C1	0.0507 (9)	0.0387 (8)	0.0430 (9)	0.0204 (7)	0.0050 (7)	0.0086 (7)
C1'	0.0452 (9)	0.0360 (8)	0.0457 (9)	0.0095 (7)	0.0024 (7)	−0.0081 (7)
C2	0.0590 (11)	0.0474 (10)	0.0607 (11)	0.0168 (8)	0.0166 (9)	0.0086 (8)
C2'	0.0483 (10)	0.0553 (10)	0.0674 (12)	0.0154 (8)	0.0129 (9)	−0.0037 (9)
C3	0.0748 (13)	0.0558 (11)	0.0598 (11)	0.0273 (10)	0.0263 (10)	0.0100 (9)
C3'	0.0676 (12)	0.0557 (11)	0.0665 (12)	0.0127 (9)	0.0307 (10)	0.0049 (9)
C4'	0.0753 (13)	0.0519 (10)	0.0533 (11)	0.0240 (9)	0.0222 (9)	0.0096 (8)
C4	0.0825 (13)	0.0497 (10)	0.0468 (10)	0.0271 (10)	0.0152 (9)	0.0020 (8)
C5'	0.0567 (10)	0.0477 (9)	0.0471 (9)	0.0220 (8)	0.0116 (8)	0.0020 (8)
C5	0.0605 (11)	0.0432 (9)	0.0475 (10)	0.0155 (8)	0.0064 (8)	0.0029 (7)
C6	0.0490 (9)	0.0393 (8)	0.0410 (8)	0.0188 (7)	0.0026 (7)	0.0066 (7)
C6'	0.0453 (9)	0.0348 (8)	0.0409 (8)	0.0102 (7)	0.0052 (7)	−0.0045 (6)
C7'	0.0491 (10)	0.0406 (9)	0.0445 (9)	0.0113 (7)	0.0044 (7)	0.0011 (7)
C7	0.0508 (10)	0.0475 (9)	0.0428 (9)	0.0210 (8)	0.0011 (7)	0.0000 (7)
C8'	0.0469 (9)	0.0474 (9)	0.0332 (8)	0.0141 (7)	0.0059 (7)	0.0049 (7)
C8	0.0479 (9)	0.0479 (9)	0.0341 (8)	0.0161 (7)	0.0033 (7)	−0.0014 (7)
C9	0.0500 (10)	0.0489 (9)	0.0485 (10)	0.0159 (8)	0.0053 (8)	0.0007 (7)
C9'	0.0503 (10)	0.0523 (10)	0.0471 (9)	0.0161 (8)	0.0081 (8)	0.0013 (8)
C10'	0.0751 (13)	0.0627 (11)	0.0629 (12)	0.0333 (10)	0.0212 (10)	0.0054 (9)
C10	0.0741 (13)	0.0480 (10)	0.0661 (12)	0.0175 (9)	0.0192 (10)	0.0059 (9)
C11'	0.0813 (15)	0.0951 (16)	0.0700 (13)	0.0506 (13)	0.0364 (11)	0.0240 (12)
C11	0.0792 (14)	0.0586 (12)	0.0743 (14)	0.0061 (10)	0.0329 (11)	0.0056 (10)
C12'	0.0585 (12)	0.0921 (16)	0.0763 (14)	0.0228 (11)	0.0333 (10)	0.0268 (12)
C12	0.0614 (12)	0.0806 (14)	0.0742 (14)	0.0183 (11)	0.0303 (10)	0.0065 (11)
C13'	0.0593 (11)	0.0573 (11)	0.0570 (11)	0.0116 (9)	0.0186 (9)	0.0129 (9)
C13	0.0610 (11)	0.0670 (12)	0.0519 (10)	0.0289 (10)	0.0151 (9)	0.0034 (9)
C14'	0.0462 (9)	0.0483 (9)	0.0460 (10)	0.0147 (7)	−0.0029 (8)	−0.0092 (8)
C14	0.0461 (9)	0.0455 (9)	0.0425 (9)	0.0164 (7)	0.0031 (7)	0.0065 (7)
C15	0.0414 (8)	0.0401 (8)	0.0355 (8)	0.0139 (7)	0.0036 (7)	0.0016 (6)
C15'	0.0407 (8)	0.0421 (8)	0.0369 (8)	0.0135 (7)	0.0026 (7)	−0.0013 (7)
C16	0.0464 (9)	0.0482 (9)	0.0335 (8)	0.0158 (7)	−0.0003 (7)	0.0011 (7)
C16'	0.0437 (9)	0.0510 (9)	0.0337 (8)	0.0140 (7)	−0.0050 (7)	−0.0063 (7)
C17'	0.0455 (9)	0.0510 (9)	0.0371 (8)	0.0216 (7)	0.0004 (7)	0.0036 (7)
C17	0.0539 (10)	0.0408 (9)	0.0370 (8)	0.0117 (7)	−0.0013 (7)	−0.0062 (7)
C18'	0.0403 (8)	0.0428 (8)	0.0340 (8)	0.0166 (7)	0.0070 (7)	0.0012 (6)
C18	0.0523 (9)	0.0369 (8)	0.0338 (8)	0.0142 (7)	0.0082 (7)	0.0017 (6)
C19	0.0597 (10)	0.0425 (9)	0.0322 (8)	0.0185 (8)	−0.0036 (7)	0.0005 (7)
C19'	0.0476 (9)	0.0442 (9)	0.0309 (8)	0.0159 (7)	−0.0012 (7)	−0.0033 (6)
C20'	0.0465 (9)	0.0442 (9)	0.0363 (8)	0.0179 (7)	0.0008 (7)	0.0022 (7)
C20	0.0528 (10)	0.0388 (8)	0.0366 (8)	0.0138 (7)	−0.0016 (7)	−0.0035 (6)
C21'	0.0526 (10)	0.0406 (8)	0.0366 (8)	0.0169 (7)	0.0008 (7)	0.0006 (7)
C21	0.0553 (10)	0.0385 (8)	0.0377 (9)	0.0166 (7)	0.0043 (7)	−0.0005 (7)
C22'	0.0698 (12)	0.0496 (10)	0.0429 (9)	0.0244 (9)	0.0103 (8)	0.0035 (8)
C22	0.0739 (12)	0.0447 (9)	0.0440 (10)	0.0209 (8)	0.0137 (9)	0.0044 (7)
C23'	0.0873 (14)	0.0550 (11)	0.0402 (10)	0.0154 (10)	0.0084 (9)	0.0052 (8)
C23	0.0924 (14)	0.0686 (12)	0.0409 (10)	0.0421 (11)	0.0132 (9)	0.0027 (9)
C24	0.0715 (12)	0.0608 (12)	0.0541 (11)	0.0329 (10)	−0.0100 (10)	−0.0186 (9)
C24'	0.0757 (13)	0.0506 (11)	0.0575 (12)	0.0139 (10)	−0.0115 (10)	0.0136 (9)
C25	0.0646 (12)	0.0419 (10)	0.0759 (14)	0.0084 (8)	0.0050 (10)	−0.0105 (9)
C25'	0.0594 (12)	0.0579 (11)	0.0772 (14)	0.0279 (9)	−0.0012 (10)	0.0115 (10)
C26	0.0668 (12)	0.0451 (10)	0.0573 (11)	0.0106 (8)	0.0177 (9)	0.0001 (8)
C26'	0.0586 (11)	0.0561 (10)	0.0527 (10)	0.0251 (9)	0.0088 (8)	0.0059 (8)
C27	0.0648 (11)	0.0332 (8)	0.0345 (8)	0.0153 (7)	0.0086 (8)	0.0032 (6)
C27'	0.0525 (10)	0.0424 (8)	0.0346 (8)	0.0240 (8)	0.0072 (7)	0.0008 (7)
C28	0.0630 (11)	0.0455 (9)	0.0478 (10)	0.0135 (8)	0.0134 (8)	0.0026 (7)
C28'	0.0509 (10)	0.0533 (10)	0.0428 (9)	0.0214 (8)	0.0111 (8)	0.0030 (7)
C29'	0.0522 (11)	0.0628 (11)	0.0547 (11)	0.0186 (9)	0.0009 (9)	−0.0072 (9)
C29	0.0694 (13)	0.0588 (11)	0.0590 (12)	0.0190 (9)	−0.0036 (10)	0.0039 (9)
C30'	0.0732 (13)	0.0727 (12)	0.0380 (9)	0.0338 (11)	−0.0020 (9)	−0.0102 (9)
C30	0.1006 (17)	0.0564 (11)	0.0393 (10)	0.0214 (11)	−0.0027 (10)	0.0063 (8)
C31	0.1025 (16)	0.0615 (12)	0.0424 (10)	0.0288 (11)	0.0252 (11)	0.0113 (9)
C31'	0.0840 (14)	0.0646 (11)	0.0393 (9)	0.0314 (11)	0.0213 (9)	0.0038 (8)
C32	0.0708 (12)	0.0565 (10)	0.0481 (10)	0.0271 (9)	0.0178 (9)	0.0086 (8)
C32'	0.0621 (11)	0.0493 (9)	0.0459 (10)	0.0165 (8)	0.0165 (8)	−0.0007 (8)
O1	0.0785 (9)	0.0500 (7)	0.0888 (10)	0.0270 (6)	0.0279 (7)	−0.0009 (6)
O1'	0.0829 (9)	0.0524 (8)	0.1026 (11)	0.0291 (7)	0.0401 (8)	0.0267 (7)
O2	0.0882 (9)	0.0573 (7)	0.0477 (7)	0.0266 (7)	−0.0055 (7)	0.0105 (6)
N1'	0.0667 (9)	0.0435 (7)	0.0328 (7)	0.0252 (7)	0.0032 (6)	0.0005 (6)
N1	0.0808 (10)	0.0384 (7)	0.0340 (7)	0.0233 (7)	0.0067 (7)	0.0010 (5)
O2'	0.1054 (11)	0.0661 (8)	0.0536 (8)	0.0357 (8)	−0.0215 (7)	−0.0226 (6)

### Geometric parameters (Å, º)

**Table:**

C1—C2	1.377 (2)	C17'—C18'	1.397 (2)
C1—C6	1.401 (2)	C17'—H17'	0.9300
C1—C14	1.503 (2)	C17—C18	1.395 (2)
C1'—C2'	1.382 (2)	C17—H17	0.9300
C1'—C6'	1.398 (2)	C18'—C19'	1.391 (2)
C1'—C14'	1.504 (2)	C18'—N1'	1.3936 (18)
C2—C3	1.379 (2)	C18—C19	1.394 (2)
C2—H2	0.9300	C18—N1	1.3957 (18)
C2'—C3'	1.378 (3)	C19—C20	1.375 (2)
C2'—H2'	0.9300	C19—H19	0.9300
C3—C4	1.368 (2)	C19'—C20'	1.373 (2)
C3—H3	0.9300	C19'—H19'	0.9300
C3'—C4'	1.371 (3)	C20'—H20'	0.9300
C3'—H3'	0.9300	C20—H20	0.9300
C4'—C5'	1.375 (2)	C21'—C26'	1.377 (2)
C4'—H4'	0.9300	C21'—C22'	1.378 (2)
C4—C5	1.377 (2)	C21'—N1'	1.4299 (19)
C4—H4	0.9300	C21—C22	1.377 (2)
C5'—C6'	1.388 (2)	C21—C26	1.380 (2)
C5'—H5'	0.9300	C21—N1	1.4246 (18)
C5—C6	1.387 (2)	C22'—C23'	1.377 (2)
C5—H5	0.9300	C22'—H22'	0.9300
C6—C7	1.491 (2)	C22—C23	1.382 (2)
C6'—C7'	1.490 (2)	C22—H22	0.9300
C7'—O1'	1.2158 (18)	C23'—C24'	1.370 (3)
C7'—C8'	1.488 (2)	C23'—H23'	0.9300
C7—O1	1.2187 (18)	C23—C24	1.366 (3)
C7—C8	1.488 (2)	C23—H23	0.9300
C8'—C13'	1.386 (2)	C24—C25	1.363 (3)
C8'—C9'	1.386 (2)	C24—H24	0.9300
C8—C13	1.381 (2)	C24'—C25'	1.364 (3)
C8—C9	1.387 (2)	C24'—H24'	0.9300
C9—C10	1.379 (2)	C25—C26	1.373 (2)
C9—H9	0.9300	C25—H25	0.9300
C9'—C10'	1.380 (2)	C25'—C26'	1.380 (2)
C9'—H9'	0.9300	C25'—H25'	0.9300
C10'—C11'	1.361 (3)	C26—H26	0.9300
C10'—H10'	0.9300	C26'—H26'	0.9300
C10—C11	1.369 (3)	C27—C28	1.373 (2)
C10—H10	0.9300	C27—C32	1.377 (2)
C11'—C12'	1.375 (3)	C27—N1	1.4335 (19)
C11'—H11'	0.9300	C27'—C28'	1.374 (2)
C11—C12	1.375 (3)	C27'—C32'	1.377 (2)
C11—H11	0.9300	C27'—N1'	1.4318 (18)
C12'—C13'	1.375 (3)	C28—C29	1.378 (2)
C12'—H12'	0.9300	C28—H28	0.9300
C12—C13	1.377 (3)	C28'—C29'	1.380 (2)
C12—H12	0.9300	C28'—H28'	0.9300
C13'—H13'	0.9300	C29'—C30'	1.370 (2)
C13—H13	0.9300	C29'—H29'	0.9300
C14'—O2'	1.2177 (18)	C29—C30	1.371 (3)
C14'—C15'	1.469 (2)	C29—H29	0.9300
C14—O2	1.2200 (18)	C30'—C31'	1.369 (3)
C14—C15	1.473 (2)	C30'—H30'	0.9300
C15—C20	1.386 (2)	C30—C31	1.367 (3)
C15—C16	1.388 (2)	C30—H30	0.9300
C15'—C16'	1.386 (2)	C31—C32	1.377 (2)
C15'—C20'	1.390 (2)	C31—H31	0.9300
C16—C17	1.371 (2)	C31'—C32'	1.380 (2)
C16—H16	0.9300	C31'—H31'	0.9300
C16'—C17'	1.364 (2)	C32—H32	0.9300
C16'—H16'	0.9300	C32'—H32'	0.9300
			
C2—C1—C6	119.21 (14)	C16—C17—C18	121.08 (14)
C2—C1—C14	117.34 (14)	C16—C17—H17	119.5
C6—C1—C14	123.34 (14)	C18—C17—H17	119.5
C2'—C1'—C6'	119.08 (15)	C19'—C18'—N1'	121.44 (13)
C2'—C1'—C14'	117.98 (15)	C19'—C18'—C17'	117.87 (13)
C6'—C1'—C14'	122.82 (15)	N1'—C18'—C17'	120.68 (13)
C1—C2—C3	121.03 (16)	C19—C18—C17	117.71 (13)
C1—C2—H2	119.5	C19—C18—N1	120.97 (13)
C3—C2—H2	119.5	C17—C18—N1	121.32 (13)
C3'—C2'—C1'	120.97 (16)	C20—C19—C18	120.62 (14)
C3'—C2'—H2'	119.5	C20—C19—H19	119.7
C1'—C2'—H2'	119.5	C18—C19—H19	119.7
C4—C3—C2	120.08 (17)	C20'—C19'—C18'	120.80 (13)
C4—C3—H3	120.0	C20'—C19'—H19'	119.6
C2—C3—H3	120.0	C18'—C19'—H19'	119.6
C4'—C3'—C2'	120.18 (17)	C19'—C20'—C15'	121.35 (14)
C4'—C3'—H3'	119.9	C19'—C20'—H20'	119.3
C2'—C3'—H3'	119.9	C15'—C20'—H20'	119.3
C3'—C4'—C5'	119.61 (17)	C19—C20—C15	121.48 (14)
C3'—C4'—H4'	120.2	C19—C20—H20	119.3
C5'—C4'—H4'	120.2	C15—C20—H20	119.3
C3—C4—C5	119.73 (16)	C26'—C21'—C22'	119.43 (15)
C3—C4—H4	120.1	C26'—C21'—N1'	119.62 (14)
C5—C4—H4	120.1	C22'—C21'—N1'	120.94 (14)
C4'—C5'—C6'	121.15 (16)	C22—C21—C26	118.69 (14)
C4'—C5'—H5'	119.4	C22—C21—N1	121.61 (14)
C6'—C5'—H5'	119.4	C26—C21—N1	119.68 (14)
C4—C5—C6	121.11 (16)	C23'—C22'—C21'	119.87 (16)
C4—C5—H5	119.4	C23'—C22'—H22'	120.1
C6—C5—H5	119.4	C21'—C22'—H22'	120.1
C5—C6—C1	118.83 (15)	C21—C22—C23	119.96 (16)
C5—C6—C7	121.35 (14)	C21—C22—H22	120.0
C1—C6—C7	119.62 (13)	C23—C22—H22	120.0
C5'—C6'—C1'	119.00 (15)	C24'—C23'—C22'	120.44 (18)
C5'—C6'—C7'	121.50 (14)	C24'—C23'—H23'	119.8
C1'—C6'—C7'	119.35 (14)	C22'—C23'—H23'	119.8
O1'—C7'—C8'	119.40 (15)	C24—C23—C22	120.77 (17)
O1'—C7'—C6'	119.77 (14)	C24—C23—H23	119.6
C8'—C7'—C6'	120.83 (13)	C22—C23—H23	119.6
O1—C7—C8	119.93 (15)	C25—C24—C23	119.42 (16)
O1—C7—C6	119.49 (15)	C25—C24—H24	120.3
C8—C7—C6	120.57 (13)	C23—C24—H24	120.3
C13'—C8'—C9'	118.59 (15)	C25'—C24'—C23'	119.82 (17)
C13'—C8'—C7'	118.81 (15)	C25'—C24'—H24'	120.1
C9'—C8'—C7'	122.48 (14)	C23'—C24'—H24'	120.1
C13—C8—C9	118.70 (15)	C24—C25—C26	120.42 (17)
C13—C8—C7	118.46 (14)	C24—C25—H25	119.8
C9—C8—C7	122.71 (14)	C26—C25—H25	119.8
C10—C9—C8	120.16 (16)	C24'—C25'—C26'	120.28 (17)
C10—C9—H9	119.9	C24'—C25'—H25'	119.9
C8—C9—H9	119.9	C26'—C25'—H25'	119.9
C10'—C9'—C8'	120.29 (16)	C25—C26—C21	120.70 (17)
C10'—C9'—H9'	119.9	C25—C26—H26	119.7
C8'—C9'—H9'	119.9	C21—C26—H26	119.7
C11'—C10'—C9'	120.51 (18)	C21'—C26'—C25'	120.13 (17)
C11'—C10'—H10'	119.7	C21'—C26'—H26'	119.9
C9'—C10'—H10'	119.7	C25'—C26'—H26'	119.9
C11—C10—C9	120.47 (17)	C28—C27—C32	119.96 (15)
C11—C10—H10	119.8	C28—C27—N1	120.04 (14)
C9—C10—H10	119.8	C32—C27—N1	119.99 (15)
C10'—C11'—C12'	119.88 (18)	C28'—C27'—C32'	119.61 (14)
C10'—C11'—H11'	120.1	C28'—C27'—N1'	119.81 (14)
C12'—C11'—H11'	120.1	C32'—C27'—N1'	120.58 (14)
C10—C11—C12	119.91 (18)	C27—C28—C29	120.11 (17)
C10—C11—H11	120.0	C27—C28—H28	119.9
C12—C11—H11	120.0	C29—C28—H28	119.9
C11'—C12'—C13'	120.29 (18)	C27'—C28'—C29'	120.05 (16)
C11'—C12'—H12'	119.9	C27'—C28'—H28'	120.0
C13'—C12'—H12'	119.9	C29'—C28'—H28'	120.0
C11—C12—C13	119.84 (18)	C30'—C29'—C28'	120.11 (17)
C11—C12—H12	120.1	C30'—C29'—H29'	119.9
C13—C12—H12	120.1	C28'—C29'—H29'	119.9
C12'—C13'—C8'	120.40 (18)	C30—C29—C28	119.70 (19)
C12'—C13'—H13'	119.8	C30—C29—H29	120.2
C8'—C13'—H13'	119.8	C28—C29—H29	120.2
C12—C13—C8	120.87 (17)	C31'—C30'—C29'	120.12 (16)
C12—C13—H13	119.6	C31'—C30'—H30'	119.9
C8—C13—H13	119.6	C29'—C30'—H30'	119.9
O2'—C14'—C15'	121.90 (15)	C31—C30—C29	120.41 (17)
O2'—C14'—C1'	119.04 (14)	C31—C30—H30	119.8
C15'—C14'—C1'	118.97 (13)	C29—C30—H30	119.8
O2—C14—C15	122.18 (14)	C30—C31—C32	120.09 (18)
O2—C14—C1	119.49 (14)	C30—C31—H31	120.0
C15—C14—C1	118.14 (13)	C32—C31—H31	120.0
C20—C15—C16	117.78 (13)	C30'—C31'—C32'	119.96 (17)
C20—C15—C14	122.31 (13)	C30'—C31'—H31'	120.0
C16—C15—C14	119.90 (13)	C32'—C31'—H31'	120.0
C16'—C15'—C20'	117.38 (13)	C31—C32—C27	119.72 (18)
C16'—C15'—C14'	120.11 (13)	C31—C32—H32	120.1
C20'—C15'—C14'	122.42 (13)	C27—C32—H32	120.1
C17—C16—C15	121.14 (14)	C27'—C32'—C31'	120.14 (16)
C17—C16—H16	119.4	C27'—C32'—H32'	119.9
C15—C16—H16	119.4	C31'—C32'—H32'	119.9
C17'—C16'—C15'	121.93 (14)	C18'—N1'—C21'	120.84 (12)
C17'—C16'—H16'	119.0	C18'—N1'—C27'	121.29 (12)
C15'—C16'—H16'	119.0	C21'—N1'—C27'	117.73 (12)
C16'—C17'—C18'	120.58 (14)	C18—N1—C21	121.97 (12)
C16'—C17'—H17'	119.7	C18—N1—C27	119.64 (12)
C18'—C17'—H17'	119.7	C21—N1—C27	117.71 (12)
			
C6—C1—C2—C3	−0.3 (2)	C14'—C15'—C16'—C17'	−175.11 (15)
C14—C1—C2—C3	176.08 (15)	C15'—C16'—C17'—C18'	1.0 (2)
C6'—C1'—C2'—C3'	0.0 (2)	C15—C16—C17—C18	2.2 (2)
C14'—C1'—C2'—C3'	175.97 (15)	C16'—C17'—C18'—C19'	−3.0 (2)
C1—C2—C3—C4	0.7 (3)	C16'—C17'—C18'—N1'	177.22 (14)
C1'—C2'—C3'—C4'	0.9 (3)	C16—C17—C18—C19	−4.6 (2)
C2'—C3'—C4'—C5'	−0.7 (3)	C16—C17—C18—N1	175.87 (15)
C2—C3—C4—C5	−0.5 (3)	C17—C18—C19—C20	2.9 (2)
C3'—C4'—C5'—C6'	−0.5 (3)	N1—C18—C19—C20	−177.47 (15)
C3—C4—C5—C6	−0.2 (3)	N1'—C18'—C19'—C20'	−177.88 (14)
C4—C5—C6—C1	0.6 (2)	C17'—C18'—C19'—C20'	2.4 (2)
C4—C5—C6—C7	175.47 (14)	C18'—C19'—C20'—C15'	0.3 (2)
C2—C1—C6—C5	−0.4 (2)	C16'—C15'—C20'—C19'	−2.3 (2)
C14—C1—C6—C5	−176.49 (14)	C14'—C15'—C20'—C19'	174.37 (15)
C2—C1—C6—C7	−175.34 (14)	C18—C19—C20—C15	1.0 (2)
C14—C1—C6—C7	8.6 (2)	C16—C15—C20—C19	−3.4 (2)
C4'—C5'—C6'—C1'	1.5 (2)	C14—C15—C20—C19	175.40 (15)
C4'—C5'—C6'—C7'	176.82 (14)	C26'—C21'—C22'—C23'	−1.8 (2)
C2'—C1'—C6'—C5'	−1.2 (2)	N1'—C21'—C22'—C23'	178.33 (15)
C14'—C1'—C6'—C5'	−176.92 (13)	C26—C21—C22—C23	−2.2 (3)
C2'—C1'—C6'—C7'	−176.66 (14)	N1—C21—C22—C23	179.60 (15)
C14'—C1'—C6'—C7'	7.6 (2)	C21'—C22'—C23'—C24'	0.9 (3)
C5'—C6'—C7'—O1'	−147.81 (16)	C21—C22—C23—C24	1.5 (3)
C1'—C6'—C7'—O1'	27.5 (2)	C22—C23—C24—C25	0.3 (3)
C5'—C6'—C7'—C8'	32.1 (2)	C22'—C23'—C24'—C25'	0.8 (3)
C1'—C6'—C7'—C8'	−152.57 (14)	C23—C24—C25—C26	−1.4 (3)
C5—C6—C7—O1	−147.02 (16)	C23'—C24'—C25'—C26'	−1.6 (3)
C1—C6—C7—O1	27.8 (2)	C24—C25—C26—C21	0.7 (3)
C5—C6—C7—C8	32.0 (2)	C22—C21—C26—C25	1.1 (3)
C1—C6—C7—C8	−153.19 (14)	N1—C21—C26—C25	179.38 (16)
O1'—C7'—C8'—C13'	28.5 (2)	C22'—C21'—C26'—C25'	1.0 (2)
C6'—C7'—C8'—C13'	−151.40 (15)	N1'—C21'—C26'—C25'	−179.12 (15)
O1'—C7'—C8'—C9'	−147.49 (17)	C24'—C25'—C26'—C21'	0.7 (3)
C6'—C7'—C8'—C9'	32.6 (2)	C32—C27—C28—C29	0.7 (2)
O1—C7—C8—C13	27.9 (2)	N1—C27—C28—C29	−177.86 (14)
C6—C7—C8—C13	−151.13 (15)	C32'—C27'—C28'—C29'	0.2 (2)
O1—C7—C8—C9	−147.83 (16)	N1'—C27'—C28'—C29'	−179.71 (14)
C6—C7—C8—C9	33.2 (2)	C27'—C28'—C29'—C30'	0.7 (2)
C13—C8—C9—C10	−1.3 (2)	C27—C28—C29—C30	0.0 (3)
C7—C8—C9—C10	174.38 (15)	C28'—C29'—C30'—C31'	−0.7 (3)
C13'—C8'—C9'—C10'	−1.4 (2)	C28—C29—C30—C31	−0.6 (3)
C7'—C8'—C9'—C10'	174.54 (15)	C29—C30—C31—C32	0.5 (3)
C8'—C9'—C10'—C11'	1.7 (3)	C29'—C30'—C31'—C32'	−0.2 (3)
C8—C9—C10—C11	1.5 (3)	C30—C31—C32—C27	0.3 (3)
C9'—C10'—C11'—C12'	−0.4 (3)	C28—C27—C32—C31	−0.8 (2)
C9—C10—C11—C12	0.1 (3)	N1—C27—C32—C31	177.71 (15)
C10'—C11'—C12'—C13'	−1.3 (3)	C28'—C27'—C32'—C31'	−1.2 (2)
C10—C11—C12—C13	−1.8 (3)	N1'—C27'—C32'—C31'	178.76 (14)
C11'—C12'—C13'—C8'	1.6 (3)	C30'—C31'—C32'—C27'	1.2 (3)
C9'—C8'—C13'—C12'	−0.2 (2)	C19'—C18'—N1'—C21'	163.18 (14)
C7'—C8'—C13'—C12'	−176.34 (16)	C17'—C18'—N1'—C21'	−17.1 (2)
C11—C12—C13—C8	1.9 (3)	C19'—C18'—N1'—C27'	−12.4 (2)
C9—C8—C13—C12	−0.4 (2)	C17'—C18'—N1'—C27'	167.36 (14)
C7—C8—C13—C12	−176.27 (16)	C26'—C21'—N1'—C18'	127.57 (16)
C2'—C1'—C14'—O2'	−98.22 (19)	C22'—C21'—N1'—C18'	−52.5 (2)
C6'—C1'—C14'—O2'	77.5 (2)	C26'—C21'—N1'—C27'	−56.7 (2)
C2'—C1'—C14'—C15'	78.33 (19)	C22'—C21'—N1'—C27'	123.21 (16)
C6'—C1'—C14'—C15'	−105.90 (17)	C28'—C27'—N1'—C18'	122.05 (16)
C2—C1—C14—O2	−106.64 (18)	C32'—C27'—N1'—C18'	−57.9 (2)
C6—C1—C14—O2	69.5 (2)	C28'—C27'—N1'—C21'	−53.67 (19)
C2—C1—C14—C15	68.51 (19)	C32'—C27'—N1'—C21'	126.39 (16)
C6—C1—C14—C15	−115.31 (16)	C19—C18—N1—C21	156.14 (15)
O2—C14—C15—C20	−165.65 (16)	C17—C18—N1—C21	−24.3 (2)
C1—C14—C15—C20	19.3 (2)	C19—C18—N1—C27	−14.2 (2)
O2—C14—C15—C16	13.1 (2)	C17—C18—N1—C27	165.40 (15)
C1—C14—C15—C16	−161.93 (14)	C22—C21—N1—C18	−41.1 (2)
O2'—C14'—C15'—C16'	9.0 (2)	C26—C21—N1—C18	140.76 (16)
C1'—C14'—C15'—C16'	−167.45 (14)	C22—C21—N1—C27	129.43 (17)
O2'—C14'—C15'—C20'	−167.53 (16)	C26—C21—N1—C27	−48.8 (2)
C1'—C14'—C15'—C20'	16.0 (2)	C28—C27—N1—C18	110.22 (17)
C20—C15—C16—C17	1.7 (2)	C32—C27—N1—C18	−68.3 (2)
C14—C15—C16—C17	−177.04 (14)	C28—C27—N1—C21	−60.5 (2)
C20'—C15'—C16'—C17'	1.6 (2)	C32—C27—N1—C21	120.97 (16)

### Hydrogen-bond geometry (Å, º)

*Cg*1 and *Cg*2 are the centroids of the C8–C13 and C27'–C32' phenyl rings, respectively.

**Table:**

*D*—H···*A*	*D*—H	H···*A*	*D*···*A*	*D*—H···*A*
C31′—H31′···O2^i^	0.93	2.51	3.419 (2)	166
C16—H16···O2′	0.93	2.44	3.219 (2)	141
C30′—H30′···*Cg*1^i^	0.93	3.00	3.894 (2)	162
C4′—H4′···*Cg*2^ii^	0.93	2.95	3.731 (2)	142

Symmetry codes: (i) −*x*+1, −*y*+1, −*z*+1; (ii) *x*, *y*−1, *z*.
